# Long-Term Ambient PM_2.5_ Exposure and Premature Mortality Across of Türkiye

**DOI:** 10.3390/toxics14080728

**Published:** 2026-08-17

**Authors:** Nebile Özmen, Volkan Duran, Fatma Şencan, Yasin Paşa, Mehmet Ali Çelik

**Affiliations:** 1 Department of Social Work, Hamidiye Faculty of Health Sciences, University of Health Sciences, Istanbul 34688, Turkey; 2 Faculty of Arts and Sciences, Iğdır University, Iğdır 76000, Turkey; volkan.duran8@gmail.com; 3 Center for Elective Courses, Istanbul Medipol University, Istanbul 34810, Turkey; fatmasencan614@gmail.com; 4 Department of Civil Engineering, İstanbul Gelişim University, Istanbul 34310, Turkey; ypasa@gelisim.edu.tr; 5 Department of Geography, Faculty of Science and Letters, Iğdır University, Iğdır 76000, Turkey; mali.celik@igdir.edu.tr; 6 Department of Geography, Nakhchivan State University, AZ 7012 Nakhchivan, Azerbaijan

**Keywords:** air pollution, PM_2.5_, attributable mortality, AirQ+, population-level analysis, Türkiye, environmental epidemiology

## Abstract

Long-term exposure to ambient fine particulate matter (PM_2.5_) is the leading environmental risk factor for premature mortality worldwide, yet comprehensive province-level evidence quantifying its health burden across Türkiye remains limited. This study investigated the spatial relationship between long-term PM_2.5_ exposure and all-cause attributable mortality across all 81 Turkish provinces in 2022 using province-level annual mean PM_2.5_ concentrations and World Health Organisation (WHO) AirQ+ estimates of PM_2.5_-attributable deaths among adults aged ≥30 years, assuming a counterfactual concentration of 5 µg/m^3^. The association between PM_2.5_ exposure and mortality was evaluated using Pearson and Spearman correlation analyses, ordinary least squares (OLS) regression, a log–log elasticity model, and population-weighted regional and exposure-quartile comparisons, while national temporal indicators for 2010–2023 were reported solely as supplementary context for the primary single-year 2022 cross-sectional analysis. The population-weighted annual mean PM_2.5_ concentration was 27.0 µg/m^3^, exceeding the WHO Air Quality Guideline by a factor of 5.4, and all 81 provinces exceeded the recommended threshold. The bivariate OLS model accounted for 41% of the between-province variation in attributable mortality rates (OLS slope = 3.23 additional deaths per 100,000 population for each 1 µg/m^3^ increase in PM_2.5_; 95% CI: 2.37–4.10; R^2^ = 0.41; *p* < 0.001), while the log–log elasticity model indicated that a 1% increase in PM_2.5_ concentration was associated with a 0.80% increase in the attributable mortality rate (95% CI: 0.65–0.95). The attributable fraction of natural-cause mortality increased progressively from 8.8% in the lowest exposure quartile to 24.6% in the highest. Nationwide, an estimated 68,440 premature deaths, representing 14.2% of all natural-cause deaths among adults aged ≥30 years, were attributable to PM_2.5_ exposure. These findings quantify a steep, spatially graded PM_2.5_-attributable mortality burden across Türkiye. As the attributable estimates derive from the WHO AirQ+ concentration–response function, the gradient describes the magnitude and spatial distribution of the modelled burden rather than an independently estimated exposure–response relationship, and on that basis the results support the adoption of WHO-aligned air-quality standards and accelerated decarbonization strategies to reduce the national health burden attributable to ambient air pollution.

## 1. Introduction

Ambient air pollution is the foremost environmental threat to human health [1]. The Health Effects Institute’s State of Global Air 2024 assessment reported that air pollution was associated with 8.1 million deaths worldwide in 2021, with more than 90% of the associated burden attributable to fine particulate matter (PM_2.5_) [2]. Long-term exposure to ambient fine particulate matter (PM_2.5_; aerodynamic diameter ≤ 2.5 μm) is consistently ranked among the leading risk factors for premature mortality by the Global Burden of Disease project [3,4]. Such particles have the ability to reach the alveoli and enter the systemic circulation, leading to oxidative stress, systemic inflammation, endothelial dysfunction and autonomic imbalance, which are pathways associated with increased risk of ischaemic heart disease, stroke, chronic obstructive pulmonary disease (COPD), lower-respiratory infection and lung cancer [5,6]. About 90% of the disease burden due to particulates is attributable to noncommunicable diseases [7,8].

The epidemiological evidence for the association of long-term PM_2.5_ exposure with all-cause and cardiopulmonary mortality is now consistent between cohort studies and meta-analyses, and suggests a generally log-linear concentration–response relationship with no apparent safe threshold [9,10]. Hazard ratios for all-cause mortality for PM_2.5_ exposure have been reported in recent harmonised analyses of over 81 million individuals in cohorts across Canada, the United States and Europe, of around 1.025–1.041 per 10 µg/m^3^ increase in PM_2.5_ exposure, with associations remaining at the lowest observed concentrations [11,12]. WHO has recently lowered its global air-quality guideline for annual mean PM_2.5_ concentration from 10 µg/m^3^ to 5 µg/m^3^ [13,14] in recognition of this growing evidence. The revision suggests that populations that were thought to be only moderately exposed actually have a significant and preventable mortality burden [15,16].

Türkiye is a case in point. The country is still highly dependent on fossil fuels, which accounted for approximately 81% of primary energy in 2022, with coal being the single largest source of electricity generation [17,18]. The average annual exposure of a person in Türkiye is about 26 µg/m^3^ of PM_2.5_, which is over five times the WHO guideline [19,20]. There is still no binding limit value for PM_2.5_ in national air-quality legislation, and monitoring of the pollutant is still not complete: less than half of the stations in the country measure PM_2.5_ and many stations do not have a high enough data completeness [21,22]. Although age-standardised rates of COPD and cardiovascular mortality have been decreasing in Türkiye over time, air pollution remains a major contributor to this mortality burden, as evidenced by independent assessments using the WHO AirQ+ tool, which have estimated that tens of thousands of deaths are attributable to air pollution each year [7,23].

Spatial aspects of the exposure–mortality relationship in Türkiye have been sparsely studied quantitatively. The industrial structure, topography, climate, and the structure of population and air quality monitoring in the 81 provinces of Türkiye vary considerably, resulting in a wide variation in exposure and baseline mortality. The policy relevance of characterising the mortality attributable to provincial PM_2.5_ and the existence of a clear and strong exposure–response gradient across provinces is of interest in targeting interventions and in justifying the adoption of WHO-aligned standards. The three main objectives of this study were as follows. First, it identified the spatial distribution of annual mean PM_2.5_ concentrations in 2022 in all 81 Turkish provinces, compared to the WHO Air Quality Guideline. Second, it measured the correlation, regression, robustness, and elasticity between provincial PM_2.5_ concentrations and PM_2.5_-attributable all-cause mortality in adults ≥30 years old. Lastly, it compared the attributable mortality burden between the seven statistical regions of Türkiye and between the four quartiles of PM_2.5_ exposure. We hypothesised that higher provincial PM_2.5_ concentrations would be associated with higher attributable mortality rates following a positive, approximately log-linear gradient that would remain robust across model specifications.

## 2. Materials and Methods

### 2.1. Study Design and Setting

**Figure 1:**
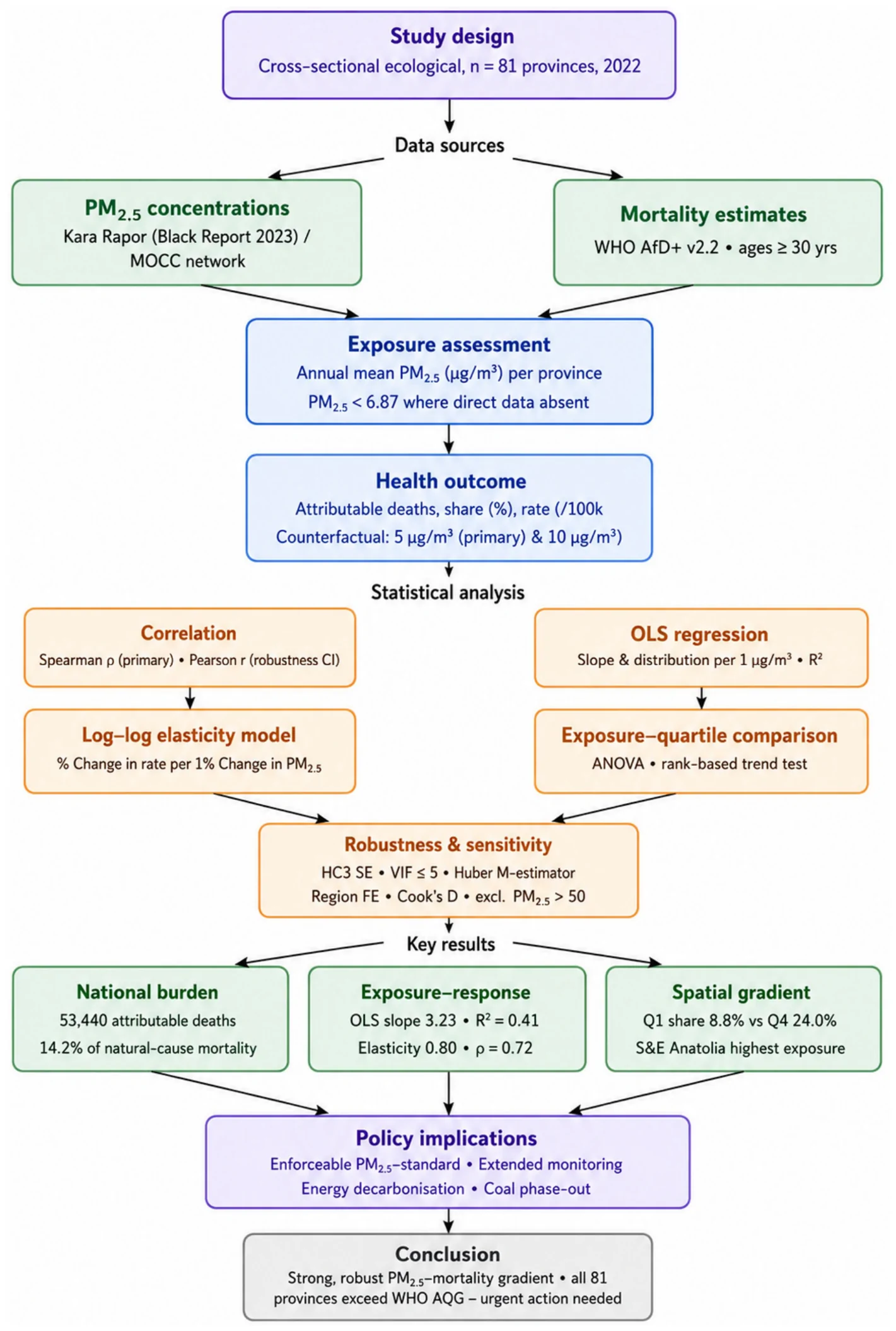
Workflow diagram of the study design and analytical framework.

We conducted a nationwide, cross-sectional population-level analysis, with the province serving as the unit of analysis. All 81 provinces of Türkiye were included for the calendar year 2022, the most recent year for which province-resolved PM_2.5_ concentrations and attributable-mortality estimates were available for the complete set of provinces. Because the analysis relies on aggregate province-level data, its findings pertain to population-level patterns and should not be interpreted as individual-level exposure–mortality associations. The overall study design, data flow and analytical framework are summarised in Figure 1.

### 2.2. Data Sources

The data for the provinces were provided by the Black Report (Kara Rapor 2024) [24], which was prepared by the Right to Clean Air Platform, a coalition of 15 Turkish health, professional and environmental organisations. The report brings together ambient air-quality data from the National Air Quality Monitoring Network (NAQMN) run by the Ministry of Environment, Urbanisation and Climate Change (MOCC), and a health-impact assessment by an academic modelling team. For each province, we extracted the annual mean PM_2.5_ concentration, the estimated number of PM_2.5_-attributable deaths, the attributable share of natural-cause deaths and the attributable death rate per 100,000 population.

Seven complementary data sources were used, each providing a different analytical layer to the population-level analytical framework (Table 1). The primary exposure metric used, PM_2.5_, was derived from the Right to Clean Air Platform (THHP), which brings together ambient air-quality measurements from the Ministry of Environment, Urbanisation and Climate Change (MOCC) National Air Quality Monitoring Network for all 81 provinces for the calendar year 2022. If PM_2.5_ was not measured directly at stations, but only PM_10_ was measured with at least 75% annual data coverage, the concentrations of PM_2.5_ were estimated by applying the WHO European conversion factor of 0.67, which is a common practice in areas with incomplete fine-particulate monitoring. Less than half of the stations in the network directly measure PM_2.5_, which is a structural limitation that can cause differential measurement error among provinces and may lead to systematic underestimation of exposure in areas where the stations are based on converted measurements. This consideration applies most strongly to the province with the highest concentration in the dataset, Hakkâri (73.8 µg/m^3^), which is served by a single city-centre station. In the source report the annual PM_10_ mean at this station is 14.5 µg/m^3^ for 2021, 110.7 µg/m^3^ for 2022 and 72.3 µg/m^3^ for 2023, a range that the report links to uncertainty in the performance of the instrument [24]. Provincial concentrations obtained by conversion are accordingly treated as estimates rather than measurements, and sensitivity analyses excluding high-exposure provinces are reported alongside the full-sample models (Section 3.6). Health-impact estimates were produced by WHO AirQ+ software (version 2.2; WHO Regional Office for Europe, Copenhagen, Denmark), with the standard log-linear concentration–response function for long-term exposure to PM_2.5_ and natural-cause mortality, parameterised with a relative risk of 1.08 per 10 µg/m^3^ increment. Attributable fractions were calculated with respect to a primary counterfactual of 5 µg/m^3^, corresponding to the WHO 2021 Air Quality Guideline (AQG), and a secondary counterfactual of 10 µg/m^3^ for comparison with previous estimates. As per the AirQ+ standard protocol for natural-cause mortality assessment, analyses were limited to adults aged 30 years and older and excluded deaths due to accidents, injuries, and COVID-19. The province-level total natural-cause death counts were algebraically calculated from the AirQ+ outputs and compared with the population-based death registration statistics provided by the Turkish Statistical Institute (TÜİK) for the years 2018–2023 to put attributable fractions into context with the observed mortality denominators. The cause-specific mortality distributions by ICD-10 chapter provided in three editions of the Ministry of Health Statistics Yearbook (2010, 2020, and 2023) allowed for temporal trend analysis of the disease groups through which PM_2.5_ has the highest mortality impact. An important comparability note: The 2010 yearbook only includes hospital deaths, while the 2018–2023 series includes population-based death registration; these two series are therefore not interchangeable for all longitudinal comparisons. Air-pollution disease burden estimates, expressed as disability-adjusted life years (DALYs) per 100,000 populations, and healthy life expectancy (HALE) at birth were obtained from the Institute for Health Metrics and Evaluation (IHME) Global Burden of Disease Study 2021 vintage. All comparisons across time were made within the GBD-2021 vintage, as values from the GBD-2019 vintage (referenced in the 2020 Ministry of Health yearbook) were not pooled with GBD-2021 estimates due to known methodological revisions between releases.

The exposure metric was the province-level annual mean PM_2.5_ concentration (µg/m^3^) for 2022. Where a station measured PM_2.5_ directly, the measured annual mean was used; where only PM_10_ was available with at least 75% data capture, PM_2.5_ was estimated from PM_10_ using a conversion factor of 0.67, as detailed in Section 2.3. Provincial means were computed as the average across qualifying stations within each province [24]. Exposure was benchmarked against the WHO 2021 annual guideline of 5 µg/m^3^ and the interim targets of 10 and 15 µg/m^3^.

The health outcome was all-cause (natural) mortality among adults aged 30 years and over, excluding deaths from accidents, injuries and COVID-19. The PM_2.5_-attributable deaths were estimated with the WHO AirQ+ software (version 2.2) based on the standard log-linear relative-risk function for long-term exposure to PM_2.5_ and natural-cause mortality, which is 1.08 per 10 µg/m^3^ increment [7]. For each province, the concentration contrast between the observed level and the counterfactual was converted to an exposure-specific relative risk using the AirQ+ log-linear function, after which the attributable fraction was calculated as AF = (RR − 1)/RR; results are presented for the primary counterfactual of 5 µg/m^3^ (the WHO guideline) and, for comparison, for 10 µg/m^3^. Three outcome measures were analysed: absolute number of attributable deaths, attributable share (per cent) of natural-cause deaths at ages 30+, and attributable death rate per 100,000 population. In this study, all-cause (natural) mortality attributable to PM_2.5_ is defined as the estimated number of excess deaths from natural (non-external) causes among adults aged ≥30 years that would not be expected under a counterfactual in which the annual PM_2.5_ concentration were reduced to the WHO guideline of 5 µg/m^3^; it therefore represents the excess deaths generated by the AirQ+ relative-risk function applied to baseline natural-cause deaths, rather than deaths from any single named disease.

Two auxiliary quantities were calculated from the published figures for each province to enable population-weighted comparisons: the total number of natural-cause deaths at ages 30+ (attributable deaths ÷ attributable share) and the population aged 30+ (attributable deaths ÷ attributable death rate × 100,000). There is a minor internal inconsistency: the totals for the provinces add up to ≈448,400 deaths, which is ~6.8% less than the authoritative national total of 480,991 deaths reported by the source. Recomputing these totals from unrounded attributable fractions alters their sum by less than 0.01%, indicating that the difference arises from the definition of the mortality denominator at province and national level rather than from the precision of the published figures. Because the province totals are back-calculated rather than published quantities, the official national figures (68,440 attributable deaths; 14.2% attributable share) are treated as primary figures, and the derived totals are used for relative comparisons of regions. Provinces were classified into the seven traditional statistical regions of Türkiye (Marmara, Aegean, Mediterranean, Central Anatolia, Black Sea, Eastern Anatolia and Southeastern Anatolia). Regional PM_2.5_ was summarised as a population-weighted mean, and the regional attributable share was pooled across constituent provinces.

### 2.3. Methods

We also created time-series indicators for three editions of the Turkish Ministry of Health Statistics Yearbook (2010, 2020 and 2023) and the attributable-death series reported in the Black Report [24] to place the 2022 cross-section in the longer-term national trends. These included: (i) the number of PM_2.5_-attributable deaths estimated by AirQ+ for 2017–2022; (ii) the distribution of deaths by ICD-10 cause group, with a particular focus on circulatory (I00–I99) and respiratory (J00–J99) diseases [18]; (iii) the air-pollution disease burden (DALYs per 100,000) from the IHME Global Burden of Disease study; and (iv) healthy life expectancy (HALE) at birth. Two comparability caveats apply and are retained throughout: the 2010 yearbook reports hospital deaths, whereas the 2018–2023 figures are population-based death-registration statistics (TÜİK); and GBD DALY estimates differ between the GBD-2019 vintage (2020 yearbook) and the GBD-2021 vintage (2023 yearbook), so values are compared only within a vintage.

Annual mean PM_2.5_ concentrations were calculated at the provincial level using data from the national air quality monitoring network. Only stations with at least 75% annual data completeness were used in the analysis to ensure data reliability. The health impact assessment was performed with the WHO AirQ+ software (version 2.2; WHO Regional Office for Europe, Copenhagen, Denmark) using a log-linear concentration–response function with a relative risk (RR) of 1.08 per 10 µg/m^3^ increase in PM_2.5_ [29]. The main counterfactual concentration was chosen as 5 µg/m^3^, as recommended by the WHO Air Quality Guidelines, and 10 µg/m^3^ was also considered in sensitivity analyses. Estimates of health burden were limited to adults ≥30 years. All variables were subjected to descriptive statistics such as mean, standard deviation, median, interquartile range (IQR) and skewness. Spearman’s rank correlation was used to assess the associations between PM_2.5_ exposure and health outcomes, and Pearson correlation coefficients with 95% bootstrap confidence intervals (10,000 resamples) were calculated as a robustness check. The regression models used were ordinary least squares (OLS), OLS with HC3 heteroscedasticity-consistent standard errors after testing for heteroscedasticity using the Breusch–Pagan test, population-weighted least squares (WLS), Huber M-estimator robust regression, regional fixed-effects models, and log–log elasticity models. Exposure–response relationships were also explored by dividing the provincial PM_2.5_ concentrations into quartiles and then conducting one-way analysis of variance (ANOVA) and non-parametric trend tests. To assess the sensitivity and robustness of the results, influential observations were identified using Cook’s distance, and the analyses were repeated after removing the provinces with annual PM_2.5_ concentrations above 50 µg/m^3^ and other influential observations to check the stability of the results.

Annual mean PM_2.5_ concentrations were estimated for each province using measurements from the national air quality monitoring network. For stations lacking direct PM_2.5_ observations, concentrations were estimated from PM_10_ according to the WHO European conversion factor [30]:

PM_2.5_ = 0.67 × PM_10_

where PM_2.5_ and PM_10_ denote the annual mean particulate matter concentrations (µg/m^3^). To ensure data quality, only monitoring stations achieving an annual data capture rate of at least 75% were included. The annual provincial mean concentration was calculated as [31]

PM_2.5,*i*_ = (1/*n_i_*) Σ*_j_* PM_2.5,*ij*_

where PM_2.5_*,i* is the annual mean PM_2.5_ concentration for province *i*, PM_2.5_*,ij* is the annual mean concentration at monitoring station *j*, and *n_i_* is the number of valid stations within province *i*.

The health burden attributable to PM_2.5_ exposure was estimated using WHO AirQ+ software (version 2.2), which applies a log-linear concentration–response model. Relative risk (RR) was assumed to increase by 8% for every 10 µg/m^3^ increase in PM_2.5_ concentration (RR_10_ = 1.08). For any concentration *C*, the exposure-specific relative risk was calculated as [32]

RR(*C*) = RR_10_^(*C* − *C*0)/10^

where RR_10_ = 1.08, *C* is the observed annual PM_2.5_ concentration, and *C*_0_ is the counterfactual concentration. The primary counterfactual concentration was defined as 5 µg/m^3^ according to the WHO Air Quality Guidelines, whereas 10 µg/m^3^ was additionally evaluated in sensitivity analyses.

The attributable fraction (AF) of mortality associated with PM_2.5_ exposure was calculated as

AF = (RR − 1)/*RR*

and the number of attributable deaths (AD) was estimated using [33]

AD = AF × *D*

where *D* represents the observed number of baseline deaths among adults aged ≥30 years. Population attributable mortality rates were subsequently expressed per 100,000 inhabitants.

Descriptive statistics were computed for all variables, including the mean, standard deviation (SD), median, interquartile range (IQR), minimum, maximum, coefficient of variation (CV), and skewness. The coefficient of variation was calculated as [34]

CV (%) = (SD/*μ*) × 100

where *μ* is the arithmetic mean.

Associations between PM_2.5_ concentrations and attributable mortality were primarily assessed using Spearman’s rank correlation coefficient (ρ), which is robust to non-normal distributions [35]:

*ρ*
= 1 − (6Σ*d_i_*^2^)/[*n*(*n*^2^ − 1)]

where *d_i_* denotes the difference between paired ranks and *n* is the sample size.

Pearson’s correlation coefficient (*r*) was additionally calculated with bias-corrected bootstrap 95% confidence intervals based on 10,000 resamples [36]:

*r*
= Σ(*x_i_* − *μ_x_*)(*y_i_* − *μ_y_*)/√[Σ(*x_i_* − *μ_x_*)^2^ Σ(*y_i_* − *μ_y_*)^2^]

Linear relationships were evaluated using ordinary least squares (OLS) regression:

*Y_i_* = *β*_0_ + *β*_1_*X_i_* + *ε_i_*

where *Y_i_* represents attributable mortality, *X_i_* denotes annual PM_2.5_ concentration, *β*_0_ is the intercept, *β*_1_ is the regression coefficient, and *ε_i_* is the random error term.

Heteroscedasticity was assessed using the Breusch–Pagan test, and when present, HC3 heteroscedasticity-consistent standard errors were applied. Additional robustness analyses included weighted least squares (WLS), where provincial population served as the weighting factor (*w_i_* = *N_i_*), robust regression based on the Huber M-estimator to reduce the influence of outliers, regional fixed-effects models to control for unobserved regional heterogeneity, and log–log elasticity models:

ln(*Y*) = *β*_0_ + *β*_1_ ln(*X*) + *ε*

where *β*_1_ represents the elasticity of mortality with respect to PM_2.5_ concentration.

To investigate potential dose–response relationships, provinces were classified into quartiles (Q1–Q4) according to annual PM_2.5_ concentration. Differences among quartiles were examined using one-way analysis of variance (ANOVA) [37]:

*F*
= *MS*_between_/*MS*_within_

while monotonic trends across quartiles were evaluated using a rank-based trend test.

Sensitivity analyses were conducted to evaluate the robustness of the findings. Influential observations were identified using Cook’s distance [38],

*D_i_* = Σ(*ŷ_j_* − *ŷ_j_*(*i*))^2^/(*p* × *MSE*)

where *p* is the number of estimated parameters, and *MSE* is the mean squared error. Provinces with Cook’s distance exceeding the conventional threshold (*D_i_* > 4/*n*) were examined individually. Furthermore, all statistical analyses were repeated after excluding provinces with annual PM_2.5_ concentrations exceeding 50 µg/m^3^ and after removing influential observations to verify the stability and robustness of the estimated associations.

Continuous variables were summarised as means, standard deviations, medians, interquartile ranges and skewness. The non-parametric Spearman rank correlation was used as the main measure of association, with Pearson correlation used as a secondary measure, with bias-corrected bootstrap 95% confidence intervals (10,000 resamples) used to complement the non-parametric measure. The linear change in the attributable death rate per unit increase in PM_2.5_ was estimated using ordinary least squares (OLS) regression. Residual variance was found to be increasing with exposure (Breusch–Pagan test, *p* < 0.001), so we also report the heteroscedasticity-consistent (HC3) standard errors, a population-weighted least squares (WLS) model, and a Huber M-estimator robust regression. High-exposure provinces (PM_2.5_ > 50 µg/m^3^) and influential provinces were excluded from sensitivity analyses, and influential observations were assessed using Cook’s distance (threshold 4/*n*) and leverage. The within-region association was estimated using a region fixed-effects model, which controlled for time-invariant structural confounding shared by provinces within the same region. A log–log model was used to estimate the exposure–response elasticity, and provinces were categorised into quartiles of PM_2.5_ by one-way ANOVA and a rank-based trend test. The attributable-share variable is, by design of AirQ+, a deterministic function of concentration, and the correlation with PM_2.5_ is therefore interpreted as a model-internal consistency check, while the attributable death rate is the substantive spatial outcome. Because that rate is the product of this attributable fraction and the province-specific baseline death rate, it is also partly determined by the concentration–response function; the regression models below should therefore be read as descriptions of how the modelled burden is distributed across provinces, not as an independent estimation of the PM_2.5_–mortality exposure–response relationship. Two-sided *p*-values < 0.05 were considered statistically significant. All analyses were conducted in Python (version 3.11.5; Python Software Foundation, Wilmington, DE, USA) using the pandas (version 2.1.4), NumPy (version 1.26.4), SciPy (version 1.11.4), and statsmodels (version 0.14.1) packages. The complete province-level dataset and all derived tables were compiled during the analysis.

## 3. Results

### 3.1. Distribution of PM_2.5_ Exposure

**Figure 2:**
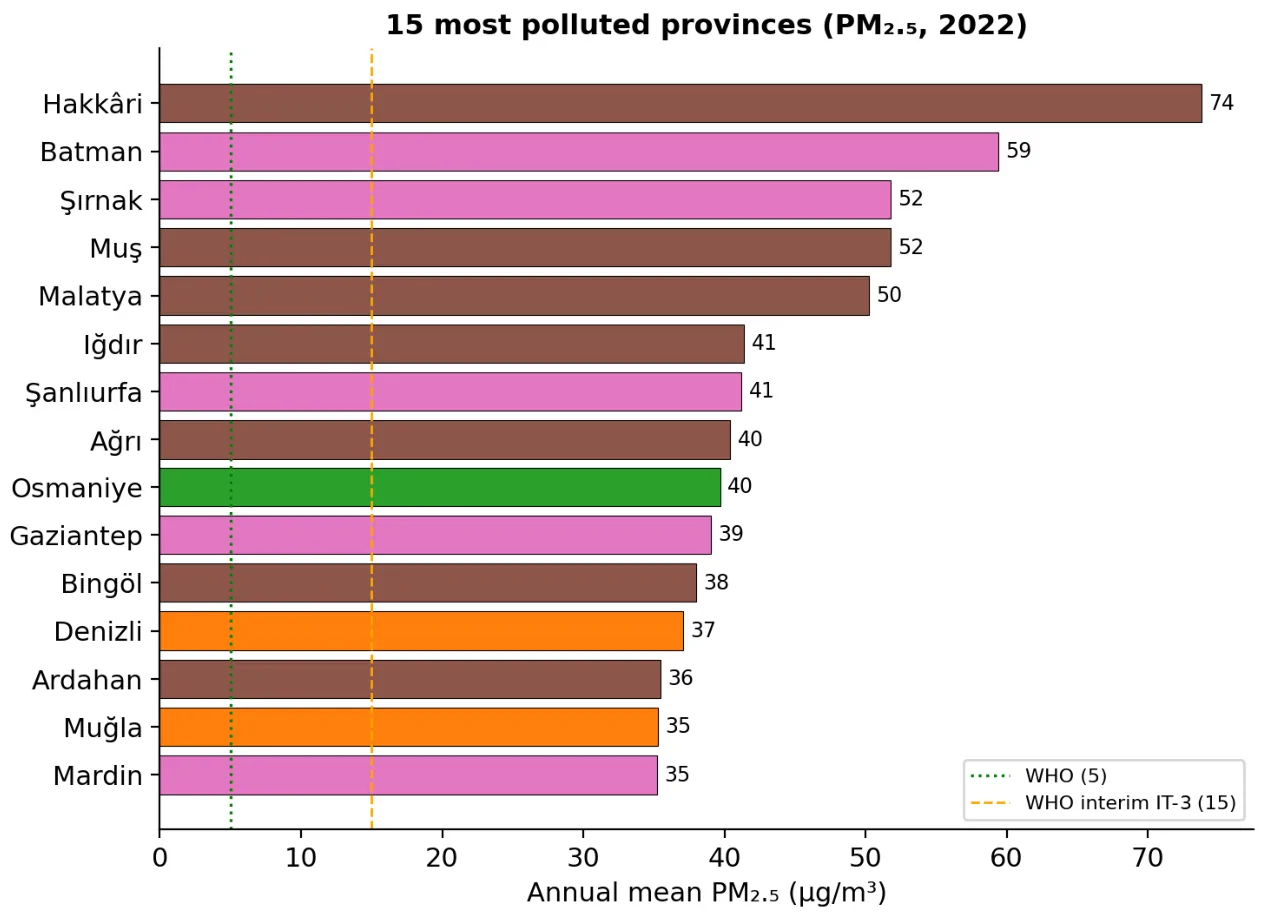
The fifteen most polluted provinces by annual mean PM_2.5_ (2022). Bars are coloured by statistical region. The dotted line marks the WHO 2021 guideline (5 µg/m^3^) and the dashed line the interim target IT-3 (15 µg/m^3^). All provinces shown exceed both. Provinces without a direct PM_2.5_ monitor are shown as PM_10_-derived estimates (Section 2.2).

The annual mean PM_2.5_ concentration in 2022 varied by more than eight times, from 9.1 µg/m^3^ in Çankırı to 73.8 µg/m^3^ in Hakkâri, with a mean of 28.1 µg/m^3^ (SD 10.8; median 26.6; interquartile range 12.7; skewness 1.4) across the 81 provinces. The population-weighted national mean was 27.0 µg/m^3^, equivalent to 5.4 times the WHO 2021 annual guideline of 5 µg/m^3^. Most importantly, all 81 provinces had levels above the guideline, with 75 (93%) above the interim target of 15 µg/m^3^ and 47 (58%) above the 25 µg/m^3^ target. The most polluted provinces were located in the eastern and south-eastern part of the country, along with some Aegean and industrial provinces (Figure 2; Table 2).

### 3.2. National Attributable Burden

An estimated 68,440 deaths among adults aged ≥30 years were attributable to PM_2.5_ exposure in 2022 under the 5 µg/m^3^ counterfactual, representing 14.2% of all natural-cause deaths in that age group (excluding accidents, injuries and COVID-19) as reported nationally by the source; the corresponding share obtained by summing the derived province totals is 15.3%. The less stringent counterfactual of 10 µg/m^3^ gave 53,545 attributable deaths (11.1%). The absolute number of attributable deaths was highest in the most populous provinces of İstanbul (8357), İzmir (4852) and Bursa (3657). The smallest absolute counts occurred in Kilis (47) and Çankırı (56); however, absolute attributable-death counts are strongly influenced by population size and should not be interpreted as rankings of exposure intensity.

### 3.3. Exposure–Response Association

**Figure 3:**
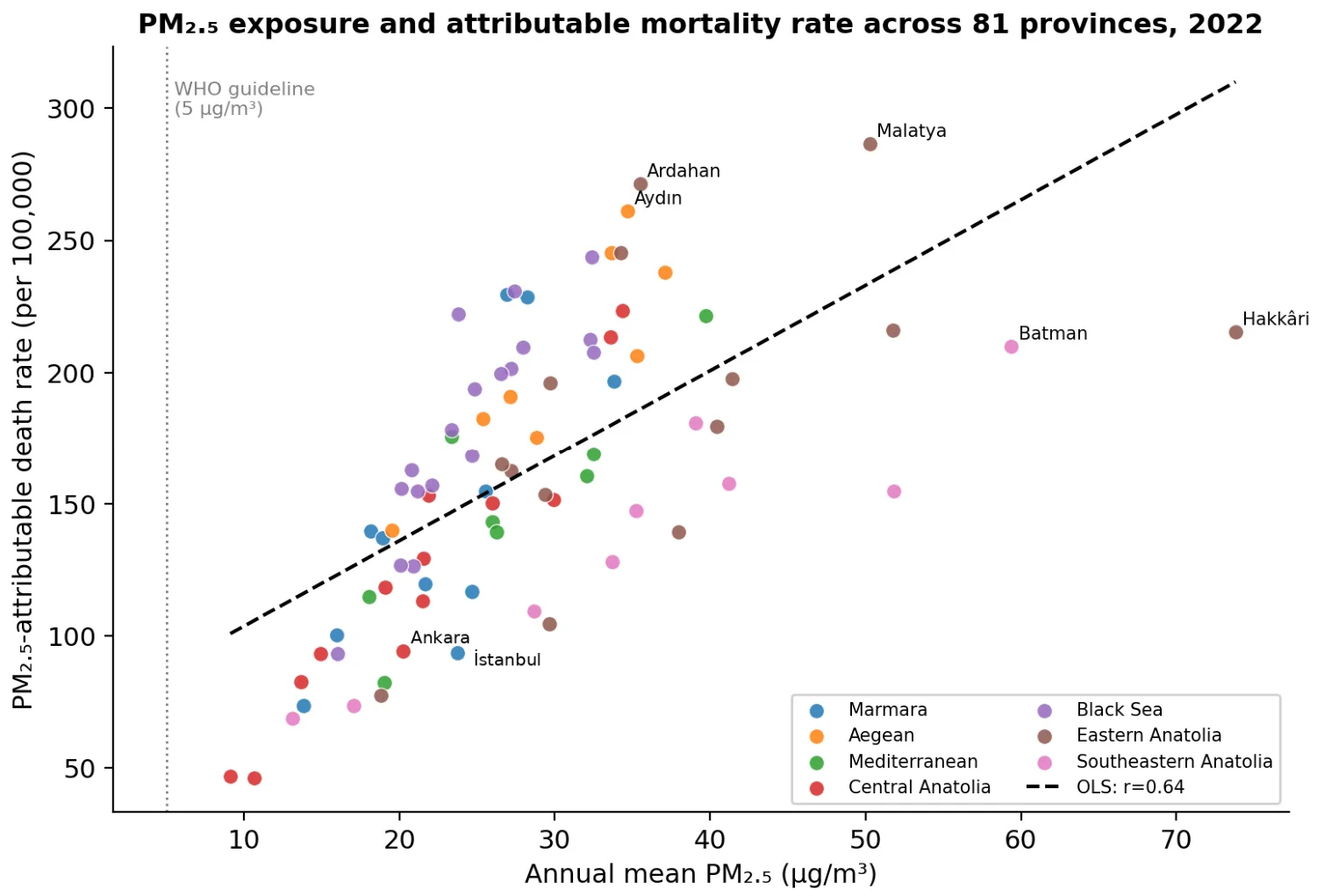
Association between annual mean PM_2.5_ and the PM_2.5_-attributable death rate (per 100,000) across the 81 provinces, 2022. Points are coloured by region; the dashed line is the ordinary least squares fit (r = 0.64). The vertical dotted line marks the WHO guideline (5 µg/m^3^). Selected provinces are labelled.

The attributable death rate per 100,000 population was strongly and positively correlated with Provincial PM_2.5_ (Spearman ρ = 0.72, *p* < 0.001; Pearson r = 0.64, bootstrap 95% CI 0.53–0.76). In the bivariate OLS model, each 1 µg/m^3^ higher provincial PM_2.5_ concentration was associated with an average of 3.23 additional attributable deaths per 100,000 population (95% CI 2.37–4.10; HC3 robust 95% CI 1.75–4.72; R^2^ = 0.41; F = 55.4; *p* < 0.001), accounting for approximately two-fifths of the between-province variation in attributable mortality rates. The log–log model showed an exposure–response elasticity of 0.80 (95% CI 0.65–0.95; R^2^ = 0.59), which suggested that a 10% increase in the provincial PM_2.5_ concentration was associated with an approximately 8% increase in the attributable death rate (Figure 3). As would be expected from the AirQ+ formulation, the attributable share of deaths was a near-perfect deterministic function of concentration (Spearman ρ ≈ 1.00; R^2^ = 0.99), which is reported as a model-internal consistency check, not as an independent epidemiological finding. The scatter of attributable death rate against PM_2.5_, with the fitted regression line, is displayed in Figure 3.

### 3.4. Dose–Response Across Exposure Quartiles

**Figure 4:**
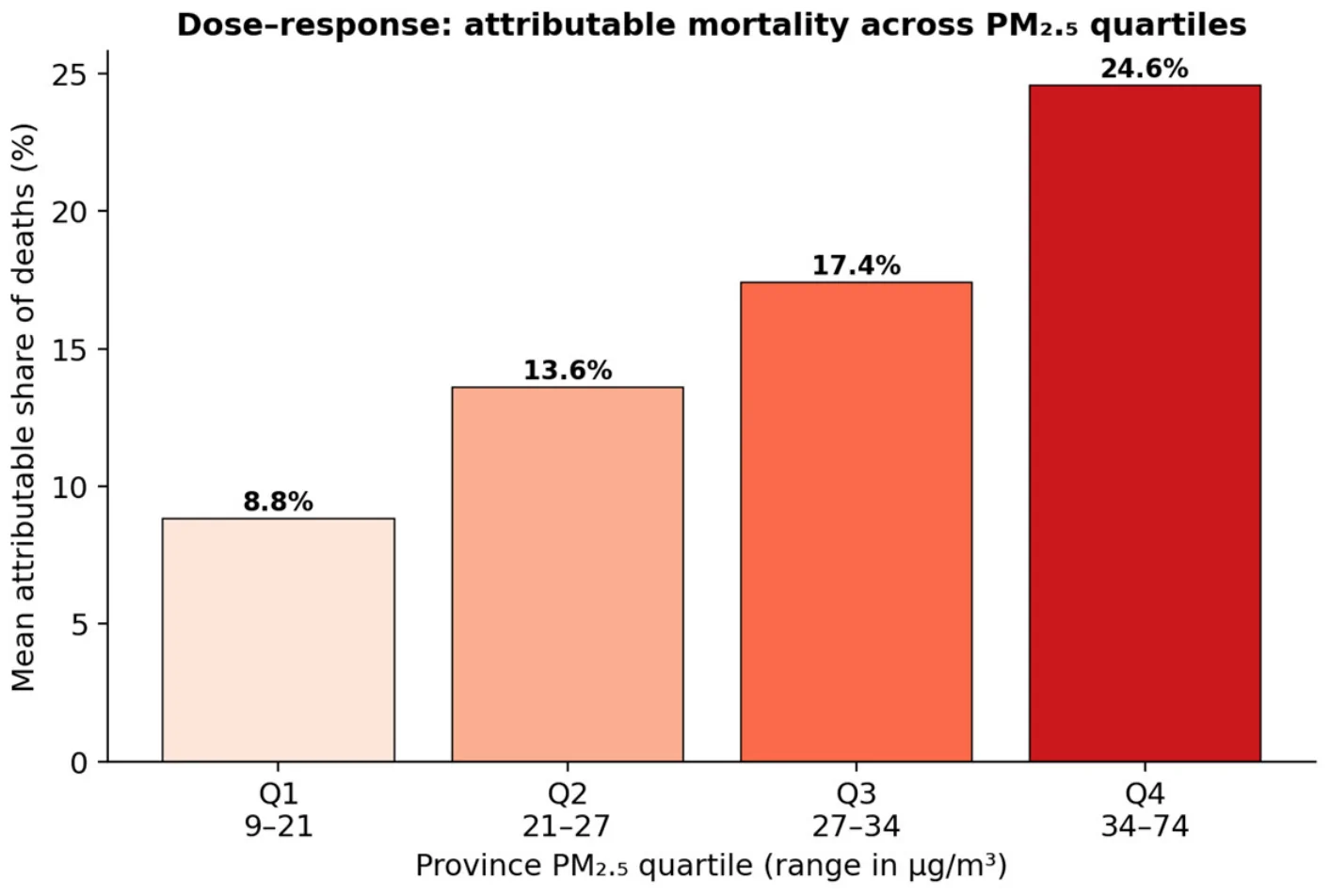
Dose–response gradient: mean attributable share of natural-cause deaths (ages 30+) across PM_2.5_ quartiles. The share rises monotonically with exposure.

A monotonic dose–response gradient was evident when provinces were grouped into PM_2.5_ quartiles (Table 3 and Figure 4). The mean attributable share of natural-cause deaths increased in a stepwise fashion from 8.8% in the lowest quartile (PM_2.5_ 9–21 µg/m^3^) to 13.6%, 17.4% and 24.6% in successive quartiles, and the mean attributable death rate rose from 102.6 to 203.9 per 100,000 (one-way ANOVA F = 29.1, *p* < 0.001; rank-based trend ρ = 0.70). The attributable mortality rate for the provinces in the highest exposure quartile was therefore almost twice that of the lowest quartile.

### 3.5. Regional Patterns

**Figure 5:**
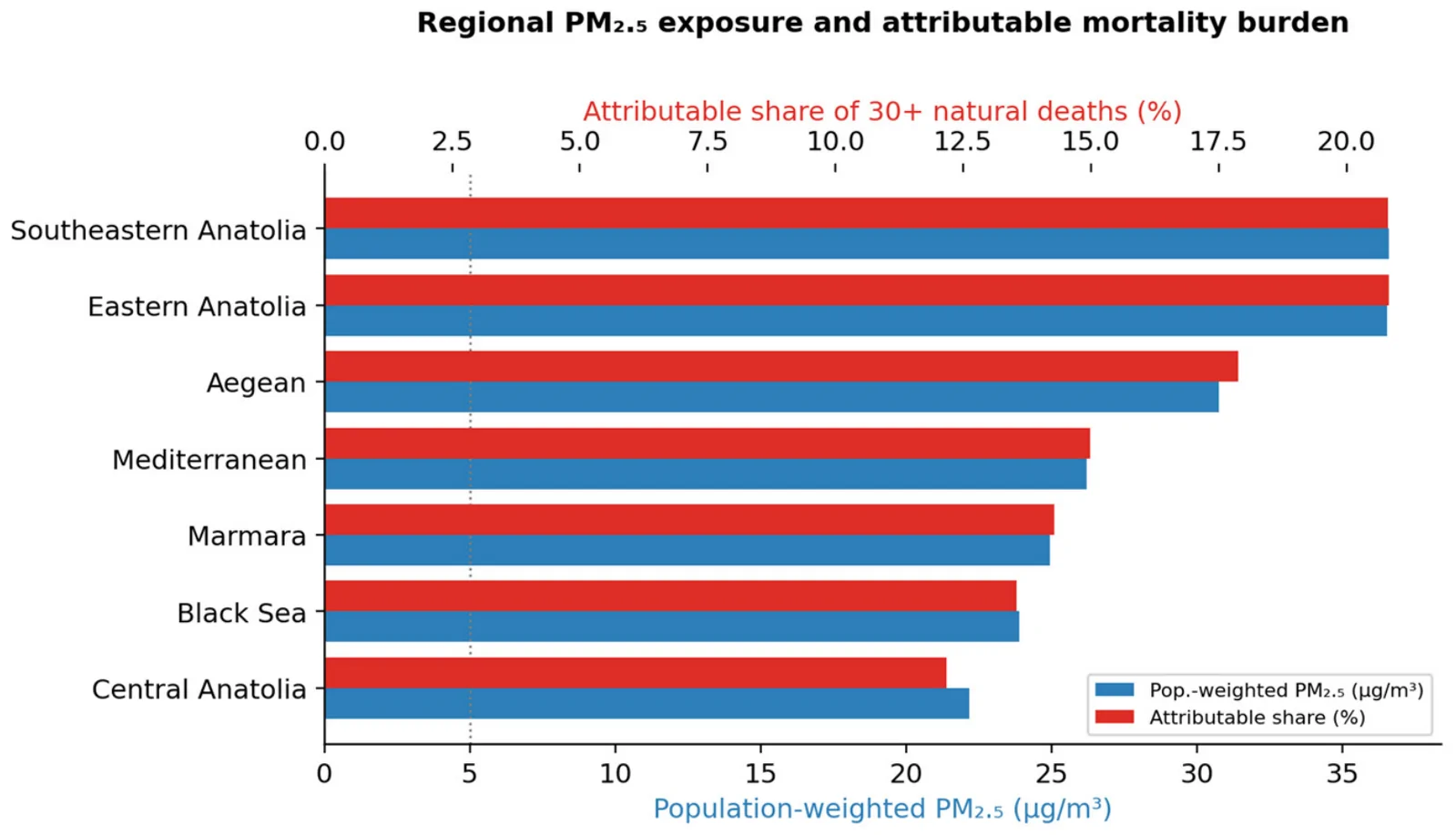
Population-weighted PM_2.5_ concentration (blue) and pooled attributable share of deaths (red) by statistical region. The dotted line marks the WHO guideline (5 µg/m^3^). This figure visualises the marked between-region differences in both PM_2.5_ exposure and attributable mortality burden across Türkiye’s statistical regions (see also Table 4).

The population-weighted PM_2.5_ burden was highest in Southeastern Anatolia (36.6 µg/m^3^) and Eastern Anatolia (36.5 µg/m^3^) and lowest in Central Anatolia (22.2 µg/m^3^) and the Black Sea region (23.9 µg/m^3^) (Table 4; Figure 5). The pooled attributable share of deaths followed the same ordering, reaching roughly 21% in the two eastern regions versus about 12% in Central Anatolia. The attributable death rate, however, was highest in the Aegean region (201.8 per 100,000) and Eastern Anatolia (181.5 per 100,000); the Aegean ranking reflects its older population age structure and higher baseline mortality, which amplify the number of deaths attributable to a given exposure. It is therefore important to distinguish absolute counts (population-driven) from rates (exposure- and age-driven): in absolute terms, the Marmara region accounted for the largest number of attributable deaths (18,676), driven by the İstanbul conurbation, despite only moderate concentrations.

### 3.6. Robustness and Sensitivity Analyses

**Figure 6:**
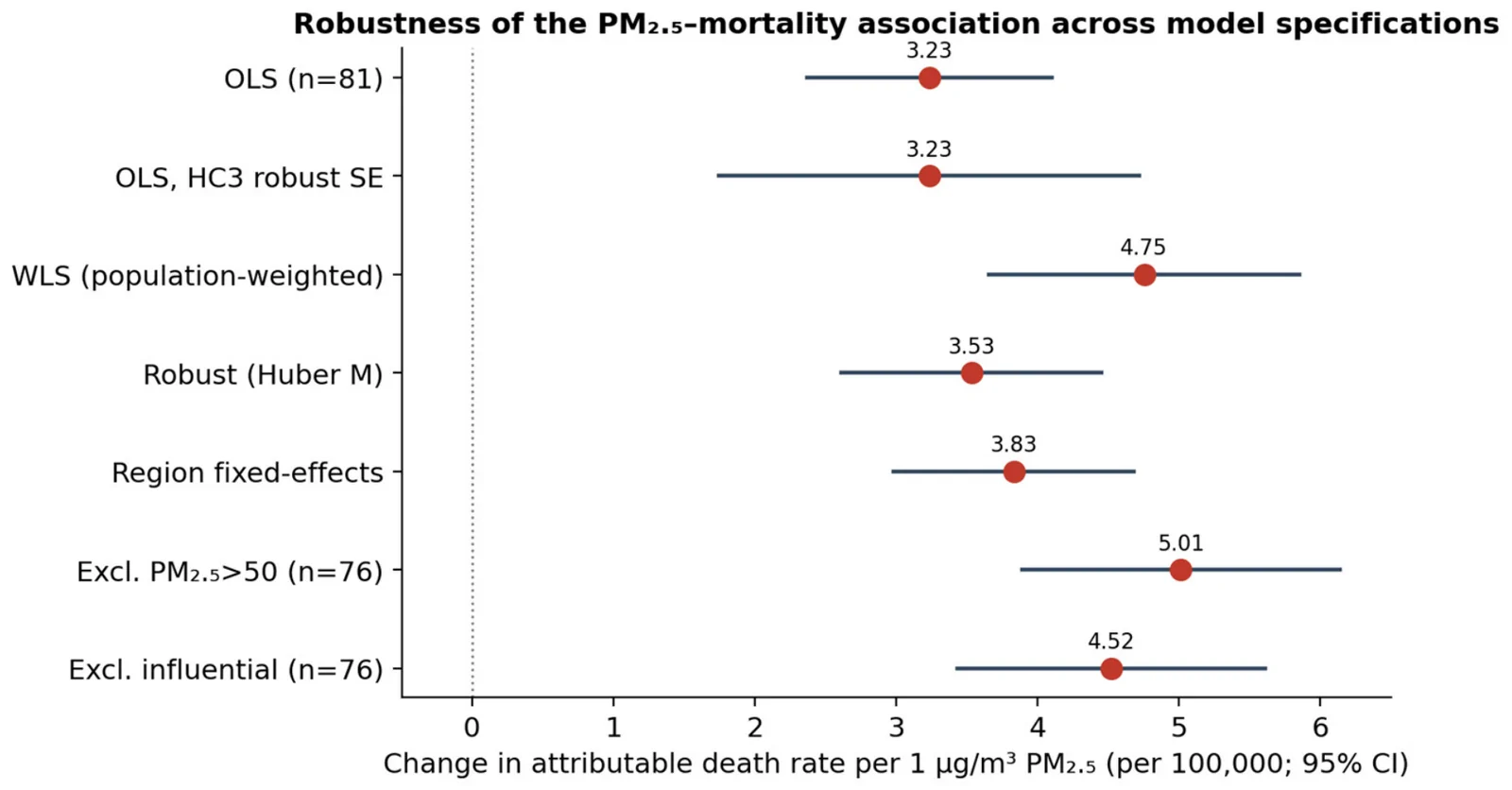
Forest plot of the estimated change in the PM_2.5_-attributable death rate per 1 µg/m^3^ increment across seven model specifications. All point estimates are positive, and all 95% confidence intervals exclude the null.

**Figure 7:**
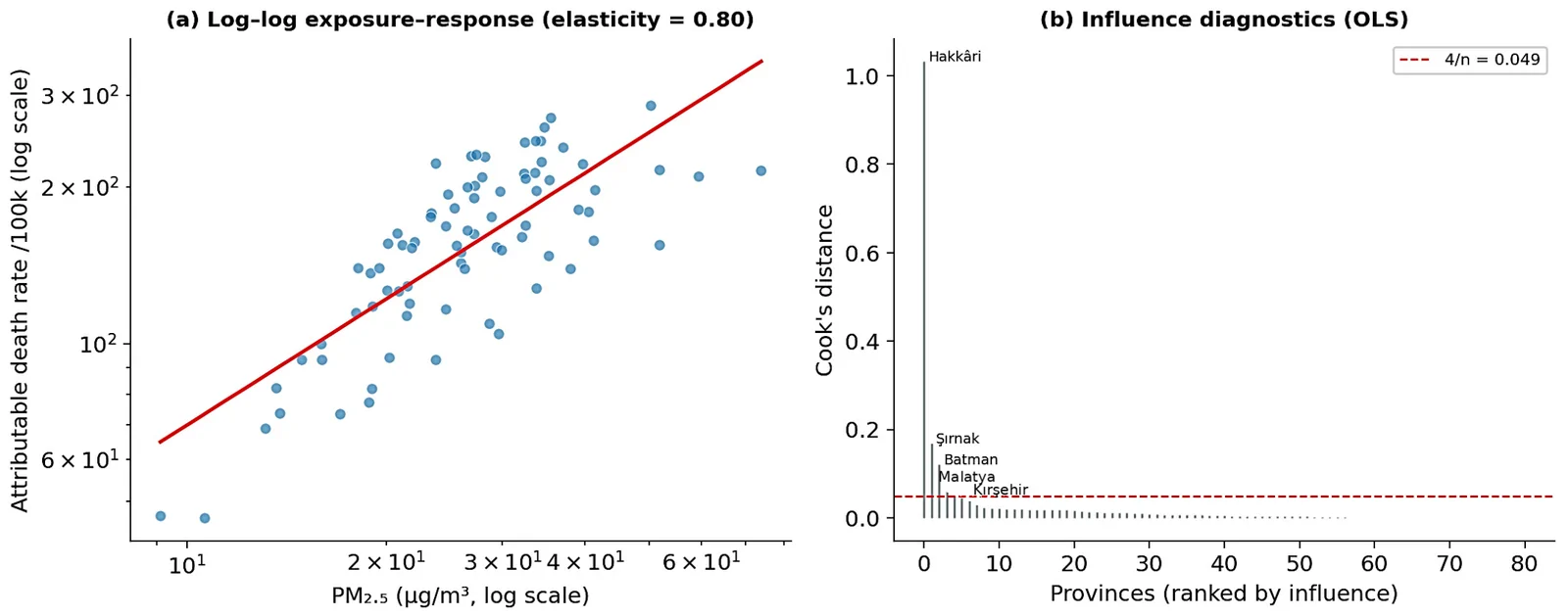
(**a**) Log–log exposure–response between PM_2.5_ and the attributable death rate (elasticity = 0.80). (**b**) Cook’s distance by province; the dashed line is the 4/*n* influence threshold. Hakkâri is the single highly influential observation; its removal strengthens the association.

The exposure–response association was robust across all model specifications (Figure 6; Table 5). Heteroscedasticity-consistent (HC3) standard errors widened but did not abolish the confidence interval (slope 3.23, 95% CI 1.75–4.72). Population-weighted least squares produced a steeper slope (4.75, 95% CI 3.65–5.85), and Huber robust regression a comparable estimate (3.53). A region fixed-effects model which removes all between-region confounding yielded a within-region slope of 3.83 (95% CI 2.98–4.68; *p* < 0.001), confirming that the association is not an artefact of broad regional differences. Hakkâri (PM_2.5_ = 73.8 µg/m^3^) was identified as the single most influential, high-leverage observation (Cook’s distance = 1.03; Figure 7b). Excluding the five provinces with annual concentrations above 50 µg/m^3^ strengthened rather than weakened the association (slope 5.01; r = 0.72; *n* = 76), as did the exclusion of the influential provinces identified by Cook’s distance (slope 4.52; r = 0.69; *n* = 76). Refitting the model with Hakkâri alone removed gave a steeper slope (3.84; 95% CI 2.89–4.79; R^2^ = 0.46; *p* < 0.001; *n* = 80), and the specification excluding all provinces above 50 µg/m^3^ is reported as the primary estimate. Across the seven specifications, every 95% confidence interval excluded the null, and point estimates ranged from 3.23 to 5.01 additional attributable deaths per 100,000 per µg/m^3^.

### 3.7. National Temporal Context (Descriptive)

**Figure 8:**
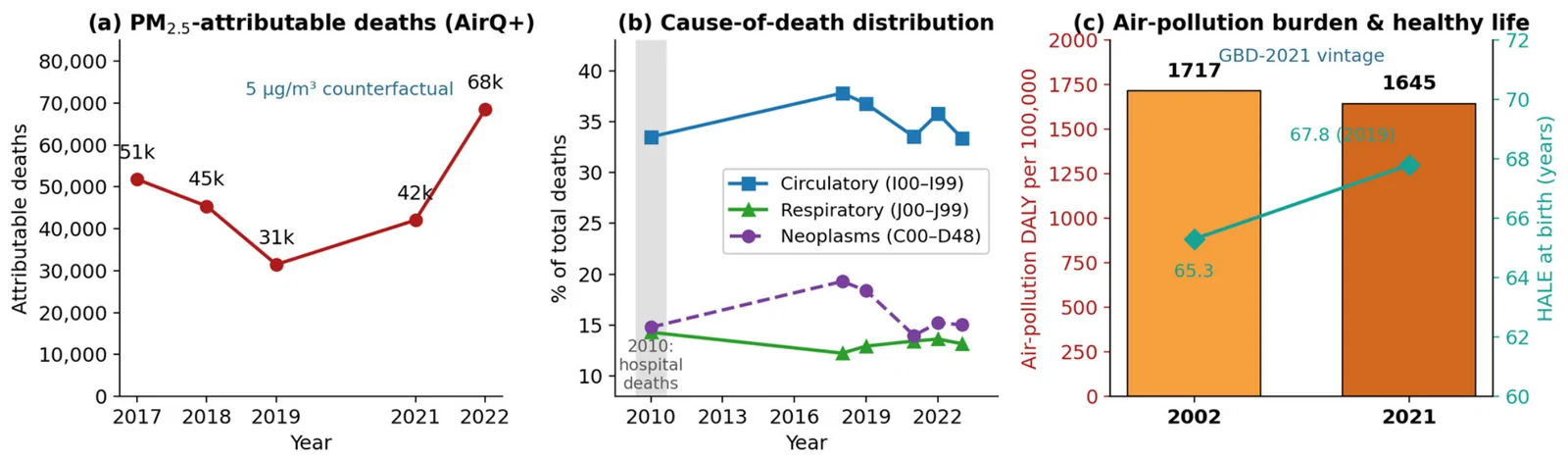
National temporal indicators. (**a**) PM_2.5_-attributable deaths estimated by AirQ+, 2017–2022 (2022 at the 5 µg/m^3^ counterfactual; earlier years at 10 µg/m^3^). (**b**) Share of total deaths from circulatory, respiratory and neoplastic causes (2010 = hospital deaths; 2018–2023 = population-based). (**c**) IHME air-pollution disease burden (DALY per 100,000, GBD-2021 vintage) and healthy life expectancy (HALE) at birth.

For descriptive context only, Table 6 and Figure 8 summarise national indicators drawn from different periods. These indicators are not part of the primary 2022 province-level analysis and are not used to infer temporal changes in the provincial PM_2.5_–mortality association. Three descriptive patterns can be noted. First, the AirQ+ estimate of PM_2.5_-attributable deaths was higher in 2022 than in 2017, although this partly reflects expanding monitoring coverage (18 provinces lacked PM_2.5_ data in 2019 versus none in 2022) and the tightening of the WHO counterfactual from 10 to 5 µg/m^3^ in 2021; on a like-for-like 10 µg/m^3^ basis the 2022 figure was 53,545. Second, circulatory and respiratory diseases the cause groups most strongly linked to particulate exposure together accounted for a stable share of all deaths across the period (circulatory 33–38%, respiratory 13–14%; jointly ≈ 47–50%). Third, within the GBD-2021 estimates, the air-pollution disease burden was lower in 2021 (1645 DALYs per 100,000 population) than in 2002 (1717), while healthy life expectancy at birth was higher in 2019 (67.8 years) than in 2002 (65.3 years). Because these indicators derive from different periods, coverage and methodological vintages, they are reported only as background context and are not interpreted as temporal changes in the province-level PM_2.5_–mortality association.

## 4. Discussion

In this nationwide population-level analysis covering all 81 provinces of Türkiye, long-term PM_2.5_ exposure was strongly and robustly associated with PM_2.5_-attributable all-cause mortality. Every province exceeded the WHO 2021 annual guideline [7]. The population-weighted mean exposure was more than five times the guideline value, and an estimated 68,440 deaths, about one in seven natural-cause deaths among adults aged 30 and over, were attributable to fine particulate pollution in a single year. The between-province exposure–response relationship was positive and graded: PM_2.5_ explained roughly 41% of the variation in attributable death rates, the fitted elasticity of 0.80 was consistent with an approximately log-linear concentration–response function, and the attributable share of deaths climbed monotonically from 8.8% to 24.6% across exposure quartiles. Crucially, the association withstood heteroscedasticity-consistent inference, population weighting, robust regression, region fixed-effects and the exclusion of influential high-exposure provinces. These findings sit comfortably within the international and national literature. Cohort studies and meta-analyses spanning diverse populations report all-cause mortality hazard ratios of approximately 1.03–1.08 per 10 µg/m^3^ of long-term PM_2.5_ [39,40,41]; the value of 1.08 per 10 µg/m^3^ applied by AirQ+ in the present analysis falls within this range, so the correspondence reflects the empirical basis of the input parameter rather than an independent validation of the estimates, and the State of Global Air 2024 attributes 8.1 million deaths globally to air pollution, the second-largest number attributable to any single risk factor, with more than 90% of them attributable to PM_2.5_ [42]. Our national estimate of 68,440 attributable deaths for 2022 is of the same order as, though somewhat lower than, a recent five-year Turkish AirQ+ assessment that reported a mean of 85,344 PM_2.5_-attributable premature deaths per year for 2019–2023 (95% CI 79,129–91,559) using a slightly broader age band (≥25 years) and a different mortality envelope [43]. Province-scale AirQ+ analyses in Türkiye, such as a recent study of Kütahya, similarly find that a meaningful fraction of all-cause and respiratory deaths could be averted by meeting WHO limit values [44]. The spatial concentration of high exposure in the east and south-east is consistent with the combined influence of residential solid-fuel combustion, topographically constrained dispersion, and industrial and transboundary sources [24].

Mechanistically, the mortality signal observed at the population level is underpinned by well-characterised biological pathways. Inhaled fine particles provoke pulmonary and systemic inflammation and oxidative stress, promote endothelial dysfunction, accelerate atherosclerosis, and perturb cardiac autonomic control and coagulation [9,45]. These processes explain why the largest share of PM_2.5_-attributable deaths globally arises from ischaemic heart disease and stroke, with COPD, lower-respiratory infection and lung cancer comprising much of the remainder [7]. Although the present analysis treats all-cause natural mortality as the outcome, which is appropriate given the systemic reach of particulate toxicity, the implied cause-specific burden in Türkiye is therefore dominated by cardiovascular disease, and GBD-based analyses confirm that ambient particulate pollution remains a leading contributor to COPD and cardiovascular mortality in the country [27]. This is directly relevant to the national non-communicable-disease agenda.

An instructive nuance emerges from the divergence between exposure and attributable-rate rankings. While the eastern regions exhibited the highest concentrations and the highest attributable shares, the highest attributable death rate per 100,000 was observed in the Aegean region. Because the attributable rate is the product of the attributable fraction and the underlying death rate, it is amplified in older, higher-mortality populations even at moderately lower concentrations. This interaction underscores that the absolute human cost of air pollution depends jointly on exposure and demographic vulnerability, and that mitigation in densely populated, demographically older regions can yield large absolute gains: İstanbul alone accounted for more than 8000 attributable deaths.

The policy implications are clear. There is no binding national limit value for PM_2.5_ in Türkiye, and the monitoring network does not fully cover the pollutant [24]. The universal exceedance of the WHO guideline reported here, combined with the steep and specification-robust exposure–response gradient, supports three specific actions: implementing an enforceable PM_2.5_ standard based on the WHO interim targets and a pathway to the guideline of 5 µg/m^3^; expanding and quality-assuring PM_2.5_ monitoring at all stations, especially in high-industry-density regions; and speeding up the transition from coal in electricity generation and residential heating. The concentration–response function is steep at the low end, and there is no threshold [46], so even partial reductions in exposure should be expected to prevent a significant proportion of the attributable burden.

These conclusions are supported by the temporal context. The structural burden of the diseases through which PM_2.5_ acts has been high and stable, with circulatory and respiratory diseases together responsible for about half of all deaths in Türkiye during 2010–2023 [25,26] and the attributable-death series sensitive to monitoring coverage and to the WHO counterfactual revision. Although the GBD air-pollution DALY rate has slightly decreased and the healthy life expectancy has gradually increased, these changes do not imply that the particulate problem has been solved; in 2022, the population-weighted national mean PM_2.5_ concentration was 5.4 times the WHO guideline, while every province exceeded the guideline value. Therefore, continued decreases in PM_2.5_ would be anticipated to translate to avoidable mortality benefits.

### 4.1. Social Policy Implications of Air Pollution

Although these social consequences were not examined directly in the present analysis, the broader literature indicates that air-pollution-related illness may have important implications for social policy. The results of this study suggest that air pollution impacts on chronic diseases and mortality require that, in the near future, not only environmental and public health policies in Türkiye should be strengthened, but also the social policy framework. Air pollution has direct impacts on the physical health of individuals, but also significant psychosocial impacts linked to chronic disease and early death. The impacts of these consequences encompass environmentally induced migration and displacement, including but not limited to climate-related displacement, unequal access to a clean and healthy environment, and the disproportionate psychosocial burden on socially disadvantaged groups, including low-income populations, people with chronic diseases, children and older adults.

Affected people are in a new disadvantaged social position, due to many chronic diseases linked to air pollution, which require social welfare and support services [47]. As older adults become more reliant on long-term care services due to chronic illnesses associated with or aggravated by long-term exposure to air pollution, for instance, there is likely to be a rise in the demand for elderly care facilities and an added workload and financial burden on social service institutions.

Climate change and environmental factors have been shown to affect human attitudes, behaviours and adaptive capacity in previous studies [48]. This means that the health effects of air pollution add to the social policy and social work systems’ responsibilities. An important priority is to increase public awareness of the relationship between environmental pollution, physical health and psychosocial well-being through effective educational and community-based interventions. In addition, social policy should include protection and support for those who are forced to move due to environmental degradation [49].

To conclude, the increasing evidence that air pollution is a social and structural determinant of health [50] will increasingly influence the design of future social policies. However, more multidisciplinary studies are needed to gain a deeper understanding of the social impact of air pollution deaths and to develop comprehensive, evidence-based social policy recommendations.

### 4.2. Strengths and Limitations

The main strengths of this study are that it covers the entire country (81 provinces) in one year, uses a standardised, internationally validated health-impact methodology (WHO AirQ+) applied to official monitoring data, and includes a thorough assessment of robustness using multiple model specifications and a transparent methodology. Several limitations should be noted. First, the analysis is based on aggregated province-level data, so the associations observed are population-level associations and cannot be used to make inferences about individual-level exposure–mortality relationships. Second, exposure was defined from fixed-site monitors and for many provinces, exposure was estimated from PM_10_ with a fixed conversion factor, which creates exposure measurement error and potential misclassification, particularly in areas with limited monitoring. This applies most strongly to the observation with the largest leverage, Hakkâri, which is served by a single station whose annual PM_10_ mean rose roughly eightfold between 2021 and 2022 and fell again in 2023 [24]. Since this observation exerts the strongest influence on the fitted slope (Cook’s distance = 1.03), the specifications excluding provinces above 50 µg/m^3^ are reported as the primary estimate, and individual province rankings at the upper end of the exposure distribution warrant corresponding caution. Third, the attributable estimates are based on the assumptions of the AirQ+ relative-risk function and counterfactual, and the attributable share is by construction a function of concentration; we therefore highlight the attributable death rate as the substantive outcome, and report the share relationship as a consistency check. The attributable death rate is itself the product of that attributable fraction and the baseline death rate, so the gradient reported here is generated by the assumed relative-risk function applied to province-specific mortality denominators and is not an independently estimated exposure–response relationship. The slope, R^2^ and elasticity should accordingly be interpreted as descriptors of how the modelled burden varies across provinces, and the seven model specifications as tests of the stability of that description rather than as replicated epidemiological evidence of causality. Fourth, there is a minor discrepancy between the derived totals and the official national denominator (~6.8%, reflecting the definition of the mortality denominator at province and national level), which makes the province-level totals approximate; the official totals are used as primary. Fifth, the cross-sectional, single-year analysis does not allow for temporal or causal sequencing, and we did not control for individual-level confounders like smoking prevalence, socioeconomic status, or healthcare access or occupational exposure, although the region fixed-effects model partially accounted for region-level structural heterogeneity; residual confounding at both the provincial and individual levels remains possible. Sixth, provinces are spatial units and may exhibit spatial autocorrelation, which may invalidate conventional independence assumptions and affect the precision of standard errors and confidence intervals. Seventh, source composition was unavailable; therefore, the analysis could not distinguish anthropogenic combustion particles from mineral dust. Because AirQ+ applies a common concentration–response function irrespective of source composition, source-specific toxicity and policy controllability were not assessed. Finally, provincial means may mask substantial within-province heterogeneity in both exposure and risk. These limitations temper causal interpretation but do not undermine the central, policy-relevant observations that every province exceeds the WHO guideline and that the modelled mortality burden is large and steeply graded across the national exposure range.

## 5. Conclusions

This nationwide population-level analysis quantifies a strong and spatially graded PM_2.5_-attributable mortality burden across all 81 provinces of Türkiye that is stable across model specifications. As these estimates derive from the WHO AirQ+ concentration–response function, the gradient describes the modelled burden rather than an independently estimated exposure–response relationship. The population-weighted national mean concentration of 27.0 µg/m^3^ was 5.4 times higher than the WHO air quality guideline; all provinces were higher than the guideline, and an estimated 68,440 deaths among adults aged 30 years and over were attributable to fine particulate pollution in one calendar year. The exposure–response gradient was similar for all analytical specifications: PM_2.5_ accounted for 41% of the between-province variance in the rates of attributable mortality, the log–log elasticity was 0.80, which is consistent with the international cohort literature, and the attributable share of natural-cause deaths increased monotonically from 8.8% in the least-exposed quartile to 24.6% in the most-exposed. Estimates remained directionally consistent across region fixed-effects, robust-regression and influential-observation sensitivity analyses; however, these analyses do not eliminate residual confounding or establish causality. Exposure intensity was also concentrated in the eastern and south-eastern provinces, but the absolute number of attributable deaths was highest in the western conurbations, especially in İstanbul Province, where more than 8000 deaths were attributable.

The policy implications are clear and pressing. There is no legally binding national limit value for PM_2.5_ in Türkiye, and less than half of the monitoring stations measure the pollutant directly, which is a combination of two structural deficits that hinder regulatory accountability and exposure assessment. The results indicate that three main actions are needed to decrease the health burden from ambient PM_2.5_ in Türkiye: implementing a national PM_2.5_ standard that meets the WHO interim targets and a legally binding roadmap to reach the 5 µg/m^3^ guideline; expanding and strengthening the national PM_2.5_ monitoring network, especially in the eastern provinces that are not well covered and where exposure levels are still high; and speeding up the phasing out of fossil fuels for electricity generation and residential solid-fuel combustion, as fossil fuels accounted for approximately 81% of Türkiye’s primary energy consumption in 2022. Where natural mineral or desert dust contributes materially to measured PM_2.5_ as may occur in some arid eastern and south-eastern provinces local source-apportionment studies should guide the balance between combustion-control and dust-mitigation measures, since not all measured PM_2.5_ is equally amenable to emission-reduction policy. The concentration–response relationship for PM_2.5_ shows no clear safe threshold, meaning that decreasing exposure to the population at any concentration level would be expected to yield measurable reductions in premature mortality, which highlights the significant public health benefits of continued improvements in air quality. The evidence base for national action will be further strengthened and these population-level estimates refined by future work using individual-level cohort data, spatial econometric models and cause-specific outcomes.

## Figures and Tables

**Table 1:** Data sources, variables extracted, methodological details, and access links for the nationwide population-level analysis of PM_2.5_-attributable mortality across 81 provinces of Türkiye.

Source	Variable(s)	Details
Kara Rapor 2024 [24] Right to Clean Air Platform (THHP), 2024	Provincial annual mean PM_2.5_ (µg/m^3^); attributable deaths; attributable share (%); death rate/100k	Consolidates MOCC National Air Quality Monitoring Network data for all 81 provinces, 2022. PM_10_-to-PM_2.5_ conversion applied (×0.67) where direct measurement unavailable (≥75% data capture required).
WHO AirQ+ v2.2 WHO Regional Office for Europe, 2022 [7]	PM_2.5_-attributable mortality estimates; attributable fraction; log-linear concentration–response function	Standard log-linear RR function for long-term PM_2.5_ and natural-cause mortality (RR = 1.08 per 10 µg/m^3^). Counterfactuals: 5 µg/m^3^ (primary) and 10 µg/m^3^. Ages ≥ 30 yrs. Excludes accidents, injuries, COVID-19.
TÜİK Death Statistics Turkish Statistical Institute, 2010–2023 [25]	Total deaths by ICD-10 cause group; circulatory (I00–I99); respiratory (J00–J99); neoplasms	Population-based death registration statistics (2018–2023). Used to derive total natural-cause deaths at ages ≥ 30 per province and for national temporal trend analysis. 2010 values are hospital deaths (not directly comparable).
Ministry of Health Statistics Yearbook Türkiye Ministry of Health, 2010/2020/2023 [26]	HALE at birth; cause-of-death distributions; health system indicators	Three editions used (2010, 2020, 2023). Healthy life expectancy (HALE) series derived from 2023 edition. Comparability caveat: 2010 = hospital deaths; 2018–2023 = population-based registration.
IHME GBD 2021 Institute for Health Metrics and Evaluation [27]	Air-pollution disease burden (DALY/100k); age-standardised mortality rates for COPD and CVD	GBD-2021 vintage used for DALY time series (2002–2021) and HALE. Values compared within vintage only; GBD-2019 (used in 2020 yearbook) not combined with GBD-2021 estimates.
WHO AQG 2021 [13] World Health Organisation, Geneva	Annual PM_2.5_ guideline (5 µg/m^3^); interim targets IT-1 (35), IT-2 (25), IT-3 (15), IT-4 (10 µg/m^3^)	Used as benchmark for exposure classification and counterfactual selection. All 81 provinces exceed the guideline; 75 exceed IT-3 (15 µg/m^3^); 47 exceed 25 µg/m^3^.
MOCC Monitoring Network Ministry of Environment, Urbanisation and Climate Change, TR [28]	Station-level PM_2.5_ and PM_10_ annual means; data capture rates	National fixed-site monitoring network. Fewer than half of stations measure PM_2.5_ directly; remaining stations report PM_10_ used for conversion. Stations with <75% data capture excluded.

**Table 2:** Descriptive statistics for PM_2.5_ exposure and PM_2.5_-attributable mortality across the 81 provinces of Türkiye, 2022 (counterfactual = 5 µg/m^3^). Attributable estimates are from WHO AirQ+ v2.2 for natural-cause deaths at ages 30+.

Variable	Mean	SD	Median	Min	Max
PM_2.5_ annual mean (µg/m^3^)	28.1	10.8	26.6	9.1	73.8
Attributable deaths (count)	845	1180	499	47	8357
Attributable share of deaths (%)	16.0	6.6	15.3	3.1	41.1
Attributable death rate (/100,000)	162.2	54.4	157.9	46.4	286.5

**Table 3:** Attributable mortality by province-level PM_2.5_ quartile, Türkiye 2022. Values are means across provinces within each quartile (counterfactual = 5 µg/m^3^).

PM_2.5_ Quartile	PM_2.5_ Range (µg/m^3^)	Provinces (*n*)	Mean Attributable Share (%)	Mean Death Rate (/100,000)
Q1 (lowest)	9.1–20.9	21	8.8	102.6
Q2	21.1–26.6	20	13.6	155.5
Q3	26.9–33.6	20	17.4	189.8
Q4 (highest)	33.7–73.8	20	24.6	203.9

**Table 4:** Regional summary of PM_2.5_ exposure and attributable mortality, ordered by population-weighted PM_2.5_ (Türkiye, 2022). Pooled share = total attributable deaths ÷ total natural-cause deaths within the region.

Region	*n*	Pop-Weighted PM_2.5_ (µg/m^3^)	Attributable Deaths	Pooled Share (%)	Rate/100k
Southeastern Anatolia	9	36.6	5482	20.8	146.8
Eastern Anatolia	14	36.5	5112	20.8	181.5
Aegean	8	30.8	13,458	17.9	201.8
Mediterranean	8	26.2	8275	15.0	136.5
Marmara	11	24.9	18,676	14.3	122.7
Black Sea	18	23.9	8311	13.5	170.1
Central Anatolia	13	22.2	9126	12.2	118.8

**Table 5:** Robustness of the PM_2.5_-attributable mortality rate association across model specifications and sensitivity analyses (outcome: attributable death rate per 100,000).

Specification	Slope (per µg/m^3^)	95% CI	*p*
OLS	3.23	2.37–4.10	<0.001
OLS with HC3 robust SE	3.23	1.75–4.72	<0.001
Population-weighted (WLS)	4.75	3.65–5.85	<0.001
Huber robust (M-estimator)	3.53	2.61–4.45	<0.001
Region fixed-effects	3.83	2.98–4.68	<0.001
Excluding PM_2.5_ > 50 µg/m^3^ (*n* = 76)	5.01	3.89–6.14	<0.001
Excluding influential provinces (*n* = 76)	4.52	3.43–5.61	<0.001

**Table 6:** National temporal indicators of air-pollution burden and related mortality, Türkiye.

Indicator	2010	2019/2020	2022/2023
PM_2.5_-attributable deaths (AirQ+)	–	31,476 (2019)	68,440 (2022)
Circulatory disease, % of deaths	33.5 *	36.8 (2019)	33.4 (2023)
Respiratory disease, % of deaths	14.3 *	12.9 (2019)	13.2 (2023)
Neoplasms, % of deaths	14.8 *	18.4 (2019)	15.0 (2023)
Air-pollution burden (DALY/100k) †	–	–	1645 (2021)
HALE at birth (years)	66.4	67.8 (2019)	–

* 2010 values are derived from hospital death records only and are therefore not directly comparable with the 2018–2023 population-based death registration statistics. † Air-pollution disease burden is expressed as disability-adjusted life years (DALYs) per 100,000 population, IHME GBD-2021 vintage.

## Data Availability

The province-level dataset supporting the conclusions of this article is provided in the maintext; any further data are available from the corresponding author on request.
